# Plasma Cell Gingivitis Treated with Photobiomodulation, with No Recurrence for a Five-Year Follow-Up

**DOI:** 10.1155/2022/2992656

**Published:** 2022-10-12

**Authors:** Federica Pulicari, Matteo Pellegrini, Maurizio Pascadopoli, Massimo Porrini, Elisabetta Kuhn, Andrea Scribante, Francesco Spadari

**Affiliations:** ^1^Maxillo-Facial and Odontostomatology Unit, Fondazione IRCCS Cà Granda Ospedale Maggiore Policlinico, Milan 20122, Italy; ^2^Department of Biomedical, Surgical and Dental Sciences, University of Milan, Via della Commenda 10, Milan 20122, Italy; ^3^Section of Dentistry, Department of Clinical, Surgical, Diagnostic and Pediatric Sciences, University of Pavia, Pavia 27100, Italy; ^4^Unit of Orthodontics and Pediatric Dentistry, Section of Dentistry, Department of Clinical, Surgical, Diagnostic and Pediatric Sciences, University of Pavia, Pavia 27100, Italy; ^5^Pathology Unit, Fondazione IRCCS Cà Granda Ospedale Maggiore Policlinico, Milan 20122, Italy

## Abstract

**Introduction:**

Plasma cell gingivitis (PCG) is a chronic inflammatory disease usually affecting the vestibular portion of the gingival mucosa. Clinical presentation is marked by erythematous macules of intense red color, confluent, and delimited from the healthy neighboring mucosa. Generally asymptomatic, the gum lesions sometimes are accompanied by burning sensations and a sense of local tension. Recommended treatment is the use of topical steroids, but with apparent initial healing that is not stable over time. The present case report concerns a patient diagnosed with PCG in November 2017, with a five-year follow-up. This is the first patient with PCG successfully treated with non-surgical periodontal therapies associated with photobiomodulation (PBM).

**Methods:**

A 64-year-old male patient had intense erythema and edema on the vestibular side of the gingival mucosa area from 1.5 to 2.5. The patient's symptomatic subjectivity parameters were evaluated through dedicated questionnaires. Erythema and gingival bleeding were also evaluated. Periodontal charting was not pathological, but intense bleeding was noted. Multiple biopsies were performed, and microscopic findings confirmed the clinical hypothesis of PCG.

**Results:**

The treatment applied was PBM associated with periodontal therapy. The patient demonstrated a progressive improvement in clinical parameters considered and reported symptoms. During the five-year follow-up, no recurrence of the disease was observed.

**Conclusions:**

The combined PBM and periodontal therapies have proved to be sufficiently effective in the control of PCG, showing reduction of the intense inflammatory, erythematous component, and gingival bleeding, and are a valid treatment alternative to topical steroids.

## 1. Introduction

Plasma cell gingivitis (PCG) is a chronic inflammatory disease affecting the gingival mucous membranes. It is a rare occurrence with an etiopathogenetic matrix still uncertain [1–3]. Microscopically, it is characterized by a dense plasma cell infiltrate. Non-neoplastic plasma cell proliferation can involve different anatomical sites, including the upper aerodigestive tract, especially the gums, such as PCG. PCGs are broadly characterized by heterogeneous morphological features, high morbidity, and a lack of a shared therapeutic approach [1, 2]. The average age of onset is 45 years and there is a higher prevalence in males [2].

Although the etiology is still unclear, many hypotheses have been postulated regarding PCG onset, ranging from trauma to hypersensitive reactions to certain types of antigens (e.g., chewing gum components, toothpaste, khat, or specific foods) or physical somatization of psychological disorders [1, 2]. However, most of these lesions are currently considered idiopathic [1, 3].

PCG characteristically affects the vestibular portions of the adherent gingival mucous membranes and then extends up to the mucogingival junction. The palatal mucosa is rarely involved. The clinical-objective features are represented by erythematous mucous membranes with intense red color, velvety, edematous, and delimited by the surrounding mucosa. These lesions are accompanied by painful symptoms, burning, and frequent bleeding [1, 4–7].

PCG presents a histopathological feature characterized by lymphoplasmacellular infiltrate near the basal membrane, spongiosis, and marked exocytosis [1–3, 5]. It is known that chronic inflammatory modifications could lead to loco-regional immunological dysregulation and induce plasma cell migration with pro-inflammatory cytokines that seem to have a key role in the immune-mediated mechanisms that trigger B-cell proliferation [2]. The diagnosis is fundamentally histopathological. In differential diagnosis, there are chronic gingivitis non-plaque dependent, atrophic erosive, bullous gingivitis, and possible plasma cell neoplastic infiltrates [3, 4, 6]. Furthermore, PCG could mimic a wide range of life-threatening entities, such as squamous cell carcinoma, autoimmune mucocutaneous bullous diseases, and lymphoproliferative disorders [1, 2, 4, 8, 9]. Plasma cell proliferation is also associated with some infectious diseases, such as syphilis [2], Castleman's disease [2, 10], primary infectious disease of the lymph node, and, recently, COVID-19 [2, 11].

The pharmacological treatment is usually symptom-related, based on local and systemic corticosteroids, immunomodulators, antibiotics, and plaque-control local mouthwashes that represent the most frequent choice as first-line therapy, to delete local irritants and to reduce immunological cell-mediated and cytokine-mediate response, preventing recurrence [1, 2, 12]. Gum improvements are always related to oral hygiene levels. However, relapses are common after stopping steroid therapy [4].

The purpose of this study is to describe a case of a patient, presented at operative unit, suffering from PCG, treated with a novel therapeutic approach based on photobiomodulation (PBM) using a laser therapy, as an alternative to pharmacological treatment, and followed up for over five years.

## 2. Case Report

### 2.1. Diagnosis and Etiology

A 64-year-old Italian male patient presented to observation at the Pathology and Oral Medicine Outpatient Clinic of Milan, in November 2017, complaining gingival bleeding.

During the collection of anamnestic data, the patient declared that he did not have allergies or voluptuous habits and that he was suffering from hypertension under treatment with nebivolol and diuretics. Following fillings of the teeth in 2016, he found erythematous hyperplastic gingivitis in the upper arch conditioning the feeding. He denied Raynaud's phenomenon, photosensitivity, lymph node swelling, weight loss, fever, xerostomia, and xerophthalmia. From the report of the specialist visit carried out by the rheumatologist, at blood tests he had isolated low anti-RNP antibody titers. Also, the patient reported no cases of psoriasis or rheumatic diseases in the family.

**Figure 1:**
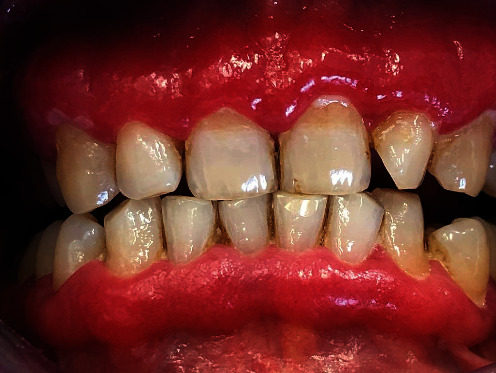
Intense edema and erythema localized to the adherent vestibular gingival mucosa, from elements 1.5 to 2.5.

The local objective clinical examination revealed intense edema and erythema localized to the adherent vestibular gingival mucosa, from elements 1.5 to 2.5 (Figure 1). The periodontal charting showed probing pocket depth <3 mm without loss of clinical attachment level, in the presence of intense bleeding. First and second level blood chemistry was prescribed and showed positivity for Proteinase 3 antibodies.

### 2.2. Treatment Objectives

The aim of this treatment is to find a therapeutic alternative for the treatment of PCG, which is effective and minimizes the risk of recurrence after the end of the treatment.

### 2.3. Treatment Alternatives

Many therapeutic treatments have been proposed over the years. Specifically, the use of topical or intralesional injection of steroids or systemic steroid therapies, contribute to the reduction of the size of the lesion, but to the suspension of the therapy do not guarantee a complete cure of the pathology [1, 2, 4]. Moreover, if the lesions are extended to the surrounding soft tissues, for example, there is the involvement of the larynx, a treatment based on chemotherapy and prednisone has been studied, favoring a temporary regression of the lesion, which recurs, however, when the therapy is suspended [1]. On the other hand, starting from the assumption that the cause of PCG is a *Candida albicans* infection, topical nystatin-based therapies have been proposed, but have been proved ineffective [1]. Another therapeutic approach indicated in the literature is low-dose radiation therapy and surgical excision. Low dose radiation caused a relative improvement in the treatment, but several studies confirm as gold standard the surgical excision by surgical blades, electrocoagulation, CO_2_ laser, or cryotherapy even with the risk of recurrence [13–15]. Finally, a therapeutic approach discussed in the literature reports a non-surgical protocol found to be effective for the management of the disease, which consists of the administration of chlorhexidine twice a day for seven weeks, home oral hygiene instructions and patient motivation, supragingival scaling, and polish, and in separate sessions complete mouth scaling with the administration of tranexamic acid for the reduction of bleeding [16].

### 2.4. Treatment Progress

**Figure 2:**
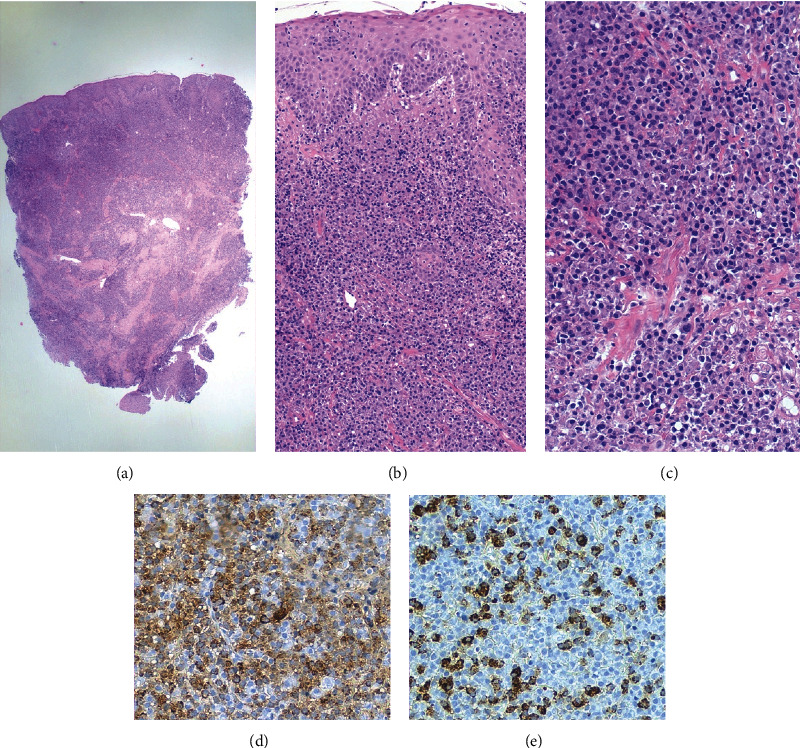
(a) The panoramic histological picture shows an oral mucosa punch biopsy, with highly cellular stroma (hematoxylin–eosin 20×). (b) At higher magnification, the oral mucosa appears spongiotic with mononuclear cell exocytosis, associated with dense stromal inflammation (hematoxylin–eosin 100×). (c) The stroma displays an intense infiltration of normal-appearing plasma cells (hematoxylin–eosin 200×). (d) Plasma cells are polyclonal, expressing both kappa (kappa light chain immunostaining 200×). (e) Lambda light chains (lambda light chain immunostaining 200×).

Two incisional biopsies were performed. The microscopic findings showed an intense inflamed infiltrate, predominantly plasmacellular, in the peripheral context of the lamina propria with edema and intra-epithelial spongiosis (Figures 2(a)–2(e)). Immunohistochemistry showed no immunoglobulin light chain restriction. The histopathological diagnosis confirmed the clinical hypothesis of PCG.

**Figure 3:**
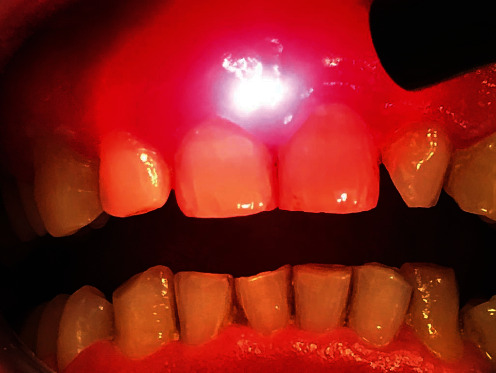
Intra-operative view, treatment performed with a diode laser, with a wavelength of 635 nm, 600-*μ*m fiber, and a handpiece with 1 cm diameter lens. A power of 300 mW was used, with continuous emission mode and with application times not exceeding 1 minute/cm^2^ (fluence 22 J/cm^2^), carrying out grid-like scanning movements.

After informed consent signature, a PBM protocol associated with oral hygiene sessions and instructions for proper oral hygiene at home was planned. PBM treatments, which began in January 2018, were performed with a diode laser (SmartFile, Deka, Calenzano (FI), Italy) with a wavelength of 635 nm, 600-*μ*m fiber, and a handpiece with a 1 cm diameter lens. A power of 300 mW was used, with continuous emission mode and with application times not exceeding 1 minute/cm^2^ (fluence 22 J/cm^2^), carrying out grid-like scanning movements (Figure 3). Application time was 12 minutes per session; 6 minutes before and 6 minutes after the hygiene session oral. The treated mucosal area was 6 cm^2^. The combined treatments were 3 times a week, for a duration of 4 weeks and a total of 12 treatments, which were completed in the middle of February 2018.

During all therapeutic phases, symptomatic subjectivities were monitored using the visual analogue or visual analogue pain scale (Visual Analogue Scale [VAS]) and the verbal numerical pain scale (Numeric Pain Rating Scale [NPRS]).

**Figure 4:**
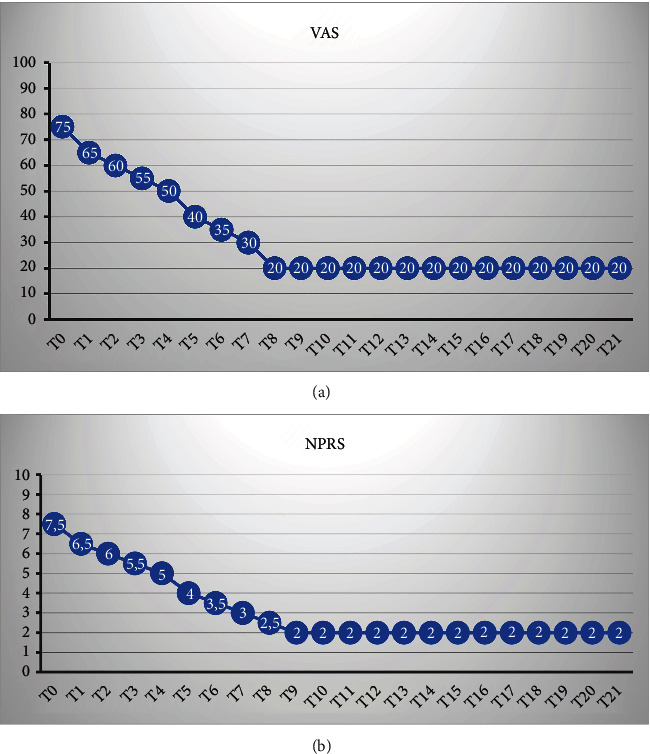
The trend of measured values over time-related to: (a) Visual Analogue Scale (VAS) and (b) Numeric Pain Rating Scale (NPRS) denotes a decrease in reported symptoms.

Periodontal indices were also evaluated with the degrees of erythema and gingival bleeding. Clinical parameters considered showed an improvement in the four weeks of PBM constant and progressive. Furthermore, there were significant symptomatic reductions found after the first two therapeutic sessions (Figures 4(a) and 4(b)).

### 2.5. Treatment Results

**Figure 5:**
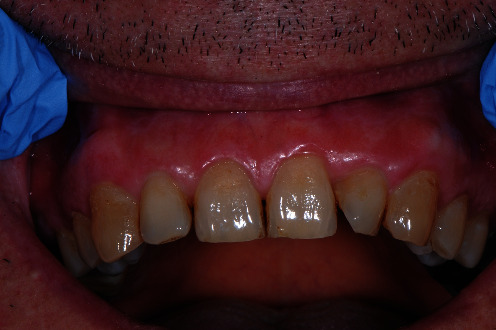
Six month after the end of treatment the mucous membranes of the lining appeared normo-chromic and normo-perfused in the absence of inflammation and symptoms.

Throughout 2018, the patient was visited monthly, without observing noteworthy relapses. A second biopsy and histopathological examination were performed about two months after the treatment ended. Histopathological examination showed a stable reduction in inflammation and plasma cell infiltrate. Then, the patient made a control visit every three months, and during the control visit in the year 2020, the blood tests were again requested, including chemistry as well as c-ANCA, p-ANCA, and IgG4 assay tests. The results of the blood chemistry tests indicated: S-Myeloperoxidase antibodies <1.0 (normal range <6.0 U/mL); Proteinase 3 antibodies: 21.8 (normally absent). Upon objective examination, the mucous membranes of the lining appear normo-chromic and normo-perfused in the absence of inflammation and symptoms (Figure 5). The patient was motivated to continue the indicated periodontal therapies and to follow scrupulous oral hygiene.

**Figure 6:**
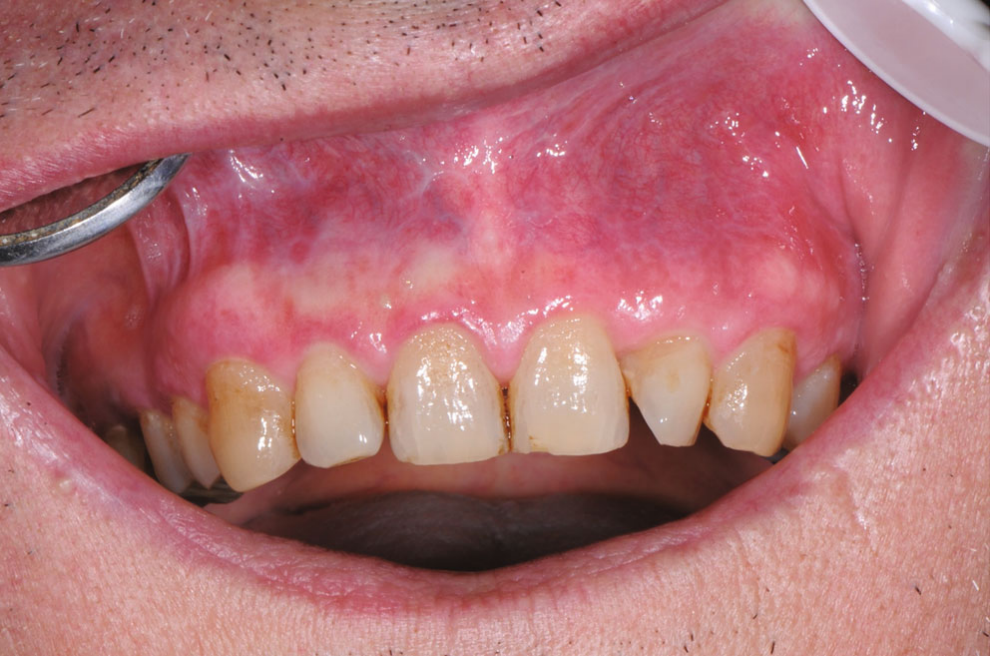
At the last check carried out in 2022, the patient's mucous membranes did not show signs of inflammation or recurrence of the original lesions.

At the last check carried out in 2022, the patient's mucous membranes did not show signs of inflammation or recurrence of the original lesions (Figure 6).

## 3. Discussion

PCG is an extended form of plasma cell mucositis of unknown etiology. Only in some patients is it possible to recognize a specific irritant cause that could favor the disease, with consequent remission of the disease after removal [1, 2, 4, 6, 7].

The role that bacterial plaque plays in the etiology of the disease is not clear. Although the lesions often appear in anatomical areas compatible with plaque-induced gingivitis, patients generally do not have significant indices of periodontal inflammation, but a general condition of periodontitis is associated [1, 2, 4, 12].

PCG is histologically characterized by a prevalent plasma cell infiltrate near the basement membrane, associated with spongiosis, and marked exocytosis [4, 6, 7, 9, 15, 17–20]. The histopathological examination is fundamental because this pathology must be placed in a differential diagnosis with chronic non-plaque dependent gingivitis, atrophic-erosive gingivitis, bullous gingivitis, and possible plasma cell neoplastic infiltrates [4, 6, 7, 9, 15, 17–20].

In our clinical experience, we wanted to test the effectiveness of PBM on tissues affected by PCG, to find a valid and lasting therapeutic alternative in the treatment of this disease. In fact, in the literature it is highlighted that laser irradiation could induce a PBM effect on cells and tissues [21, 22], influencing modulation of cell behaviors, and enhancing the processes of tissue repair. PBM could also induce cell proliferation and stem cell differentiation. In addition, laser therapy has an analgesic and anti-inflammatory effect on the treated tissues [21, 22]. It could be interesting in the future to test PBM in combination with ozone therapy [23], probiotics [24], postbiotics [25], and other natural compounds [26] in order to understand if it would be possible to improve tissue repair.

In the illustrated case report, in the clinical evaluations, the parameters of symptomatic subjectivity of the patient were considered through the administration of the VAS and NRS scale, finding a progressive improvement of subjective symptoms. At histological examination, a substantial decrease in plasma cell inflammatory infiltrate in the peripheral context of the lamina propria was found after PBM treatment. To date, after five years of treatment, the patient presents healthy gingival tissue, without noteworthy recurrence of the lesion, with no need of topical steroid therapy.

## 4. Conclusion

Our case is the first to show the efficacy of PBM treatment with diode lasers in containing PCG, and, above all, the lack of side effects. The use of laser light, capable of inducing PBM effects, has proved to be a valid alternative to pharmacological treatments, which are not stable over time. Indeed, PBM treatment and supportive periodontal therapies have been found to be effective in reducing inflammatory components and gingival bleeding. Importantly, the therapeutic results obtained remained stable for a follow-up period of five-year, without any additional pharmacologic therapy.

## Data Availability

The authors confirm that the data supporting the findings of this study are available within the article.
